# Reverse Correlating Love: Highly Passionate Women Idealize Their Partner’s Facial Appearance

**DOI:** 10.1371/journal.pone.0121094

**Published:** 2015-03-25

**Authors:** Gul Gunaydin, Jordan E. DeLong

**Affiliations:** 1 Department of Psychology, Bilkent University, Ankara, Turkey; 2 Department of Psychological and Brain Sciences, Indiana University—Bloomington, Bloomington, Indiana, United States of America; Zhejiang Key Laborotory for Research in Assesment of Cognitive Impairments, CHINA

## Abstract

A defining feature of passionate love is idealization—evaluating romantic partners in an overly favorable light. Although passionate love can be expected to color how favorably individuals represent their partner in their mind, little is known about how passionate love is linked with visual representations of the partner. Using reverse correlation techniques for the first time to study partner representations, the present study investigated whether women who are passionately in love represent their partner’s facial appearance more favorably than individuals who are less passionately in love. In a within-participants design, heterosexual women completed two forced-choice classification tasks, one for their romantic partner and one for a male acquaintance, and a measure of passionate love. In each classification task, participants saw two faces superimposed with noise and selected the face that most resembled their partner (or an acquaintance). Classification images for each of high passion and low passion groups were calculated by averaging across noise patterns selected as resembling the partner or the acquaintance and superimposing the averaged noise on an average male face. A separate group of women evaluated the classification images on attractiveness, trustworthiness, and competence. Results showed that women who feel high (vs. low) passionate love toward their partner tend to represent his face as more attractive and trustworthy, even when controlling for familiarity effects using the acquaintance representation. Using an innovative method to study partner representations, these findings extend our understanding of cognitive processes in romantic relationships.

## Introduction

There has long been interest in passionate love—an intense state of romantic attraction [1] associated with reward-related activation in the brain [2–4], physiological arousal [5], and mental preoccupation with the partner [6]. Another defining feature of passionate love is idealization—evaluating romantic partners in an overly favorable light [7–9], an idea also reflected in the famous Shakespeare quote "love looks not with the eyes, but with the mind." But, no research to date has examined how passionate love is linked with *visual representations* of the partner. Specifically, do women who are passionately in love represent their partner’s facial appearance more favorably—as more attractive, trustworthy, or competent—compared with women who are less passionately in love? To address this question, the present research used the reverse correlation technique—an innovative method borrowed from cognitive psychology—to reveal mental representations of romantic partners [10].

Mental representations of close relationship partners—including those of romantic partners—are considered the building blocks for interpersonal cognition [11–13]. They consist of detailed information about specific relationship partners including episodic memories, partners’ traits and attitudes, expectations about how they would behave in various situations as well as their visual appearance [14,15]. This rich information about relationship partners has profound influences on social cognition; they strongly color person perception [16], affective responses [17], and their regulation [18]. How we represent relationship partners also has profound influences on our well-being, with individuals who represent their partners more favorably experiencing greater physical and mental health [19,20]. Despite their known importance, very little is known about *visual* aspects of partner representations and factors predicting such representations.

One factor that can be expected to color mental representations of romantic partners is passionate love. Idealizing romantic partners or evaluating partners in an overly favorable light is thought to go hand in hand with feelings of passionate love [7]. For example, Tennov’s [9] interviews with hundreds of individuals who were passionately in love suggested that when madly in love, individuals tend to exaggerate their partners’ virtues and downplay their faults. Based on this work, passionate love should be expected to affect how individuals represent their partner and should result in idealizing the partner’s facial appearance. However, past work on idealization has primarily focused on relationship quality and satisfaction instead of passionate love, and showed that individuals experiencing greater satisfaction [21] and relationship quality [22] reported that their partner has favorable characteristics—such as being kind and affectionate. Thus, the link between passionate love and partner representations remains relatively unexplored.

The present research fills this important gap by using the reverse correlation technique, which can be used to reveal how individuals and social categories are represented [10,23]. This technique asks participants to complete a classification task in which they are presented with two faces superimposed with noise and are asked to select the face that resembles a certain target individual (e.g., a newly-met acquaintance) or a social category (e.g., Moroccans). Averaging across noise patterns in faces selected as resembling the target individual or category approximates the visual representation of the target. To obtain quantifiable assessments of visual representations obtained in the classification task, a separate group of participants are asked to evaluate these representations on various traits (e.g., trustworthiness). For example, using these procedures Dotsch and colleagues [24] showed that greater implicit prejudice about stigmatized groups is associated with representing these groups less favorably—as less trustworthy and more criminal-looking. Although this work suggests that the reverse correlation technique can be used to reliably capture visual representations, only one study to date used this method to study how relationship processes affect social cognition. This study looked at how romantic relationship status influences visual representations of potential mates and found that women who are in a romantic relationship (vs. single) tended to represent a newly encountered opposite-sex man as less attractive [25]. However, the reverse correlation technique has not been used to study how individuals represent the physical appearance of their romantic partner.

### Present Research

How do women who are passionately in love picture their partner’s facial appearance in their mind? Do they represent their partner more favorably compared with women who are less passionately in love? To address this question, we used reverse correlation techniques comprised of a classification phase and a rating phase. In the classification phase, we asked heterosexual women in a within-participants design to complete two forced-choice classification tasks in counterbalanced order: one for their romantic partner and one for a male acquaintance who is roughly the same age as the partner and whom participants knew for as long as they knew their partner. Assessing the representation of an acquaintance as such helped control for familiarity effects. On each trial of the classification task, participants viewed two faces: one created by combining structured, sinusoidal noise to a morphed average male face (i.e., base face) and the other by subtracting the same noise from the base face. Participants were asked to select the face that most resembled the target individual (partner or acquaintance).

Participants were divided into two groups based on their passionate love score (high passion vs. low passion). Classification images for each group were calculated by averaging across noise patterns selected as resembling the partner or the acquaintance and superimposing the averaged noise on the base face. In the rating phase, a separate group of women evaluated the classification images (of romantic partners and acquaintances) on the three basic traits underlying social judgments—attractiveness, trustworthiness, and competence [26] to look at whether high (vs. low) passion women represent their partner’s facial appearance more favorably.

## Materials and Methods

### Participants

Forty-six heterosexual women (*Mean age* = 21.3, *SD* = 1.5) in a romantic relationship (relationship length = 6–81 months) completed the classification phase. Based on past work [24,27], we aimed to collect data from fifty participants. Our stopping rule was to collect data until the end of the semester or until 50 participants were recruited, whichever is sooner. We stopped data collection at the end of the semester, resulting in a sample size of 46. Recruiting only female participants in the current study allowed us to use a single base face in the reverse correlation paradigm. The results obtained using this paradigm are highly dependent on the base face used in a given study because the visual representation of the target individual is derived by altering the base face in accordance with each participant’s responses [23]. Therefore, for heterosexual individuals, studying representations of the romantic partner necessitates the use of an opposite-sex base face. Recruiting both men and women in the current study would require using different base faces for participants from each sex (i.e., a male base face for female participants and a female base face for male participants). Given past work used a single base face in the reverse correlation paradigm to derive visual representations [10,23–25,27,28], we recruited only women in the current study following Karremans and colleagues [25], who studied relationship processes using female participants.

In the rating phase, we again recruited female participants to make sure that standards with which these participants used to evaluate male faces would be similar to participants who completed the classification task. An Internet sample of women reached via email communication completed this phase. The sample size was estimated a priori using G*Power [29]. The sample size to achieve. 80 power at *p*<.05 assuming Cohen’s *d*_*z*_ = .30 was estimated as 90. At the end of each day, we checked if the number of participants who completed the online survey exceeded this number. Two hundred and forty-six women (and ten men who were not eligible to participate) accessed the online survey and 93 women (*Mean age* = 21.7, *SD* = 1.4) completed it. Eight of these participants failed to complete one or two of the twelve possible trustworthiness, attractiveness, and competence ratings, resulting in degrees of freedom ranging from 88 to 92. We obtained similar results as those reported in the results section when we repeated all analyses after estimating missing data using multiple imputation.

All research was approved by the Institutional Review Board at Bilkent University and was conducted in Ankara, Turkey. All participants provided written consent, either by signing an informed consent form or clicking on a button in a written online consent form.

### Procedure

**Fig 1:**
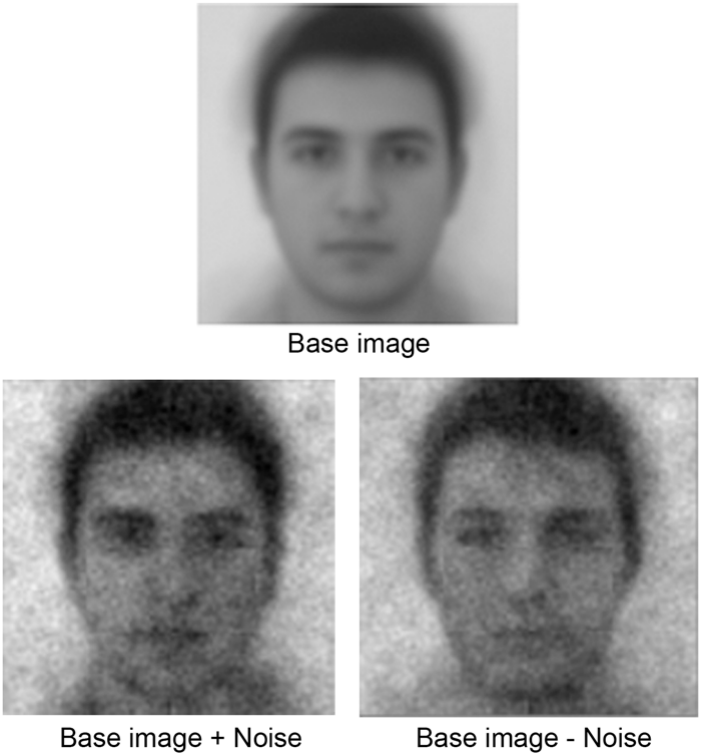
Stimuli Used in the Reverse Correlation Paradigm. Base image and an example of pairs of faces presented on a trial of the classification task.

In the classification phase, mental representations were measured by asking participants to complete a forced-choice classification task comprised of 300 trials. To create the base face used in the task, fifty male faces from a standardized database [30] were averaged using morphing software (PsychoMorph) [31]. To minimize high-frequency edge effects, the base image (a 512x512 pixel image) was run through a light Gaussian blur with 15-pixel radius using open-source image manipulation software [32] (Fig. 1). The individual male faces used to derive the base image are available by request from Dr. Baris Ozener at bozener@cumhuriyet.edu.tr.

The classification task was programmed using Matlab with Psychophysics Toolbox-3 extensions [33]. On each trial of the task, participants viewed a pair of faces. Each pair was constructed by combining the base image with a bank of 4,092 sinusoids constructed to cover different sizes, cycles, phases, and orientations in accordance with Matlab code written for a previous study and made available online [24]. The amount of contrast for each sinusoid in the bank was randomly generated for each trial, resulting in a randomly generated face that can be described by a 4,092 element vector containing contrasts between-1 and 1. Each randomly generated face was paired with its mathematical opposite, a face generated from the same randomly generated vector but with the sign flipped, resulting in emphasis of sinusoids that are deemphasized in the paired face and vice versa (Fig. 1).

In one version of the classification task, participants were asked to select the face that most resembled their romantic partner. They repeated the same task for a male acquaintance who is roughly the same age as their partner (*M*_partner_ = 22.91, *SD*_partner_ = 3.10; *M*_acquaintance_ = 23.41, *SD*_acquaintance_ = 6.06; *t*(46) = .67, *p* = .508) and whom they knew for as long as they knew their partner (*M*_partner_ = 40.01 months, *SD*_partner_ = 26.89; *M*_acquaintance_ = 43.64 months, *SD*_acquaintance_ = 41.50; *t*(46) = .72, *p* = .477). The order of tasks measuring partner and acquaintance representations were counterbalanced across participants. To encourage participants to visualize the target individual vividly, prior to each classification task participants were asked to describe and rate the facial appearance of their partner (or acquaintance). After completing the classification tasks, participants completed the Passionate Love Scale [7] using a 7-point (*Not at all true* to *Definitely true*) scale (*Cronbach’s α* = 0.88, *M* = 5.87, *SD* = 0.80).

**Fig 2:**
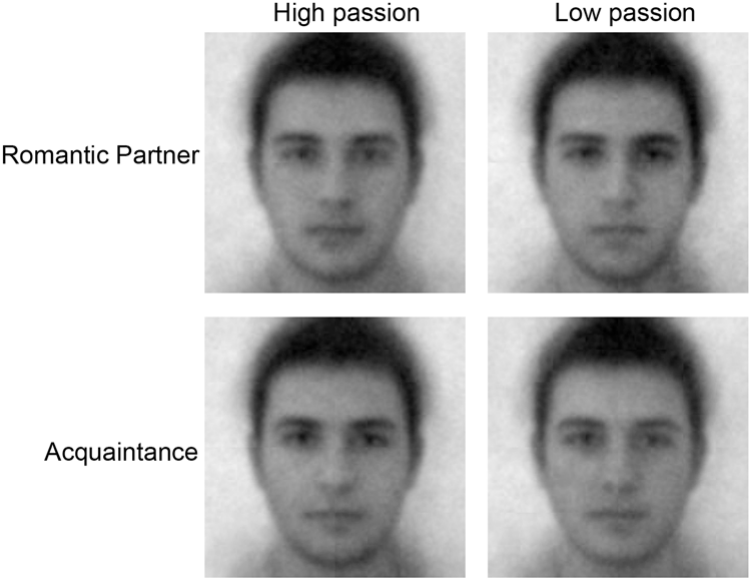
Classification Images. Partner and acquaintance classification images of women experiencing high vs. low passionate love.

Participants were divided into high passion and low passion groups via median split. Relationship duration of high-passion (*M* = 26.17 months, *SD* = 21.09) and low-passion groups (*M* = 28.27 months, *SD* = 18.20) did not significantly differ, *t*(44) = .36, *p* = .720. Classification images for each group were computed by collecting the contrast vectors for faces selected as resembling the partner or the acquaintance, averaging the contrast vectors separately for high passion and low passion women, and then constructing a face by combining the list of contrasts with the bank of sinusoids and mixing them with the base face (95% user generated sinusoid, 5% base face). The resulting classification images shown in Fig. 2 provide an approximation of how high passion and low passion women represent facial appearance of their partners and acquaintances [23]. To investigate whether the favorableness of classification images (i.e., visual representations) vary across high vs. low passion groups, we needed to quantify trustworthiness, attractiveness, and competence of these classification images. To achieve this, we used a rating phase in which evaluations of classification images were obtained from a separate group of women following past work using the reverse correlation paradigm [23–25,27,28].

In the rating phase, a separate group of women was asked to evaluate the classification images of high passion and low passion groups on attractiveness, trustworthiness, and competence using a 7-point (*Not at all* to *Very*) scale. The images and the traits were presented in random order. Raters’ relationship status was not associated with evaluations of classification images (all *p*s>.34), nor did it moderate the effect of passionate love on evaluations of these images (all *p*s>.46).

## Results

Partner classification images of high (vs. low) passion individuals were rated as more trustworthy (*M*_high-passion_ = 4.18, *M*_low-passion_ = 3.15, *t*(92) = 5.96, *p*<.001, 95% CI [.69, 1.38], *d* = .62), attractive (*M*_high-passion_ = 3.51, *M*_low-passion_ = 2.98, *t*(91) = 3.49, *p* = .001, 95% CI [.23, .84], *d* = .36), and competent (*M*_high-passion_ = 4.02, *M*_low-passion_ = 3.41, *t*(91) = 3.96, *p*<.001, 95% CI [.30, .91], *d* = .41) by a separate group of participants. This indicates that high-passion individuals tend to possess idealized representations of their partner’s facial appearance.

**Fig 3:**
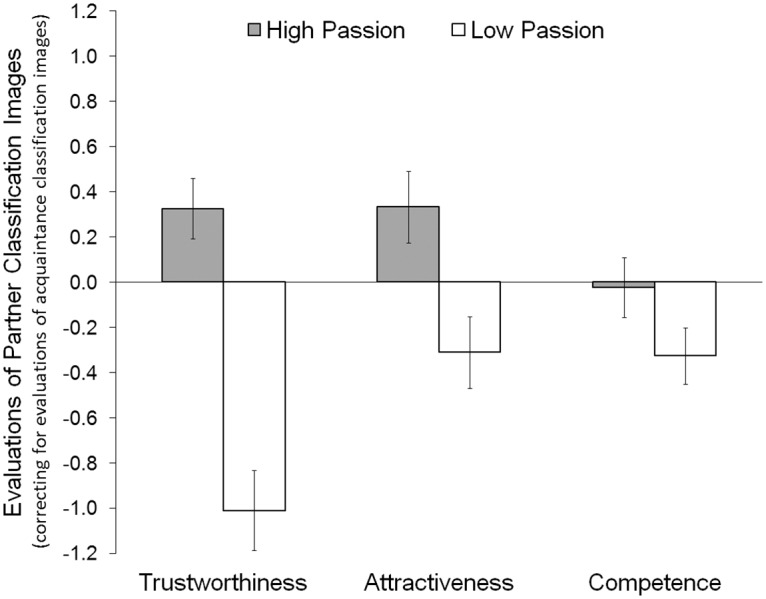
Evaluations of Partner Classification Images by a Separate Group of Women. To control for familiarity, evaluations of acquaintance classification images were subtracted from evaluations of partner images. Positive values indicate that partners are represented more favorably than acquaintances, negative values indicate that partners are represented less favorably than acquaintances, and zero indicates that partners and acquaintances are represented equally favorably. Error bars represent standard errors of the mean.

Do women who are passionately in love view all familiar others through rose-colored glasses or are these effects specific to romantic partners? To control for familiarity effects, we subtracted ratings of acquaintance classification images from those of partner classification images for high-passion and low-passion groups. If the results hold after controlling for ratings of acquaintance images this would indicate that the link between passionate love and partner representations cannot be accounted by familiarity. This was indeed the case. Controlling for familiarity, partner images of high (vs. low) passion women were still judged as more trustworthy, *t*(91) = 5.38, *p*<.001, 95% CI [.84, 1.83], *d* = .56, and attractive, *t*(89) = 2.79, *p* = .006, 95% CI [.19, 1.10], *d* = .29 (Fig. 3). However, partner images of these two groups no longer significantly differed in competence, *t*(88) = 1.57, *p* = .120, 95% CI [-.08, .69], *d* = .17.

## Discussion

Using reverse correlation techniques for the first time to study partner representations, the present study showed that women who feel high (vs. low) passionate love toward their partner tend to represent their partner’s face more favorably. Even after accounting for familiarity effects using the representation of a highly familiar acquaintance, high (vs. low) passion women represented their partner as more attractive and trustworthy. Interestingly, partner representations of high and low passion women did not appreciably differ in competence after accounting for familiarity effects. These findings suggest that women who are passionately in love do not indiscriminately represent their partners more favorably. But they rather emphasize characteristics that are most central to romantic relationships—trustworthiness and attractiveness—in visual representations of their partner, consistent with research showing that defining features of passionate love are trust and sexual attraction [34].

Using the reverse correlation technique to study partner representations in the present study conferred several advantages. First, this method is less susceptible to biases associated with self-reports of partner evaluations typically employed by past work [21]. That is, when asked explicitly how trustworthy or attractive their partner is, an individual feeling strong passionate love might be more motivated to paint a favorable picture of their partner. Rather than asking participants to report their evaluations of their partner, the present research asked participants to select the face that most resembled their partner—a method likely to reduce self-report biases. Second, visual representations uncovered by the reverse correlation technique were shown to map onto implicit evaluations [24]. Given implicit evaluations of romantic partners were found to reliably predict relationship longevity even controlling for explicit measures such as relationship satisfaction [35], partner representations revealed by the reverse correlation technique might be good predictors of relationship outcomes. Thus, the novel way of studying partner representations employed in present research has the potential to guide future research on relationship well-being and longevity.

The findings raise important questions regarding theorizing on stress-buffering effects of activating significant other representations. Past work has shown that activating mental representations of romantic partners help reduce stress created by enduring physical pain [18,36] and recalling upsetting memories [20]. Importantly, these stress-buffering effects were most pronounced for individuals who represent their partners as trustworthy and dependable [20]. Given that women feeling greater passionate love were shown in present work to have more favorable partner representations it is possible that stress-buffering effects of activating partner representations might be more pronounced for high-passion individuals. Therefore, although speculative, reminding couples of the times when they felt most passionate toward their partner might help reduce stress in day-to-day life and in therapeutic settings.

The present findings also have implications for theorizing on the evolution of love {Gonzaga, 2008, The evolution of love and long-term bonds}[37], which proposed that love evolved to help solve important reproductive challenges—keeping partners together long enough to increase chances of reproduction and chances that the resulting offspring will receive adequate care to survive till reproductive age. Our findings suggest that a novel mechanism by which the reproductive benefits of passionate love are realized might be through idealizing the partner’s facial appearance. Women who are passionately in love with their partner represent their partner’s face as more trustworthy and attractive, in which case they will be motivated to stick with their partner long enough to increase chances of reproduction and offspring survival.

Given the current study recruited only women to study the relationship between passionate love and partner representations, an interesting direction for future research is to look at whether this relationship would generalize to men. On the one hand, based on theorizing suggesting women compared with men are more sensitive to relational cues [38], one might expect that the association between passionate love and partner representations would be more pronounced in women compared with men. On the other hand, past work showed that both men and women idealize their romantic partner’s personality [21], suggesting that the association between passionate love and idealizing partner’s facial appearance might hold for both men and women.

Another important direction for future research is to use experimental designs to complement the correlational design used in current work, which would help clarify the direction of the causal relationship between passionate love and partner representations. It is possible that passionate love leads to idealizing partner’s facial appearance. However, it is also plausible that women whose partners are more attractive and trustworthy end up feeling greater passionate love. Future work can experimentally manipulate passionate love [39] to uncover whether momentary feelings of passion toward one’s partner would increase the positivity of the partner representation.

The present study uncovered for the first time how passionate love is linked with visual representations of romantic partners, extending past work on passionate love and idealization. The results showed that women who are passionately in love tend to idealize their partner’s facial appearance—representing him as more attractive and trustworthy—than women who are less passionately in love, even when accounting for familiarity effects. Thus, when it comes to feelings of passionate love Shakespeare seems to be right in his claim that "love looks not with the eyes, but with the mind."
